# Towards adult vaccination in India: a narrative literature review

**DOI:** 10.1080/21645515.2019.1682842

**Published:** 2019-12-02

**Authors:** Resham Dash, Ashish Agrawal, Vasant Nagvekar, Jayesh Lele, Alberta Di Pasquale, Shafi Kolhapure, Raunak Parikh

**Affiliations:** a GSK, New Delhi, India; b GSK, Mumbai, India; c Lilavati Hospital & Research Centre, Mumbai, India; d Indian Medical Association, National Hospital Board of India, Mumbai, India; e GSK, Wavre, Belgium

**Keywords:** Adult immunization, India, guidelines, immunosenescence, barriers, life-course immunization, vaccination coverage

## Abstract

Despite vast improvements in childhood vaccination coverage in India, adult vaccination coverage is negligible. Our aim was, therefore, to create awareness about the importance of adult immunization. Although the true burden of vaccine-preventable diseases (VPDs) among Indian adults is unknown, adults are particularly vulnerable during outbreaks, due to a lack of immunization, waning immunity, age-related factors (e.g. chronic conditions and immunosenescence), and epidemiological shift. There are no national adult immunization guidelines in India, and although several medical societies have published adult immunization guidelines, these vary, making it unclear who should receive which vaccines (based on age, underlying conditions, etc.). Other barriers to adult immunization include vaccine hesitancy, missed opportunities, and cost. Steps to improve adult vaccination could include: adoption of national guidelines, education of healthcare providers and the public, and promotion of life-course immunization. Improving adult vaccine coverage could help reduce the burden of VPDs, particularly among older adults.

## Introduction

India is the second most populous country in the world, with nearly 1.4 billion people, accounting for approximately 18% of the world population.^1^ It has been estimated that up to 63 million people in India are pushed into poverty by health expenses each year.^2^ Further, while 15% of households faced significant health costs in 2004–2005, this rose to 18% in 2011–2012.^3,4^ Although the burden of communicable, maternal, neonatal, and nutritional (CMNN) diseases is declining in India, they still accounted for approximately one third of disability adjusted life years and around one quarter of deaths in 2016.^5^ Even though the burden of CMNN diseases was considerably higher among children than adults, among those aged ≥15 years (representing approximately 72% of India’s population in 2017^6^), CMNN diseases still accounted for 14–30% of disability adjusted life years and 17–29% of deaths (depending on age group).^5,7^

The discovery of antibiotics in the early twentieth century and the eradication of smallpox in 1979 via vaccination were major breakthroughs in the fight against infectious diseases.^8^ However, despite the huge beneficial impact of antibiotics, antibiotic resistance has become one of the greatest threats to global health.^9^ Further, the risk of infection has increased greatly with mutation, globalization, and increased travel.^8,10^ Therefore, infectious diseases continue to be a major worldwide public health problem^8,11^ and vaccine-preventable diseases (VPDs) affect many thousands of adults worldwide, resulting in high morbidity, mortality, and economic burden.^12^ Furthermore, unvaccinated adults can spread diseases to infants who have not yet been immunized (e.g. pertussis).^13,14^ Therefore, improving adult vaccination coverage could have positive consequences for individuals, families, and communities. Adult vaccination could also help to reduce healthcare costs,^12^ which is particularly important among poorer communities.

**Figure 1.**
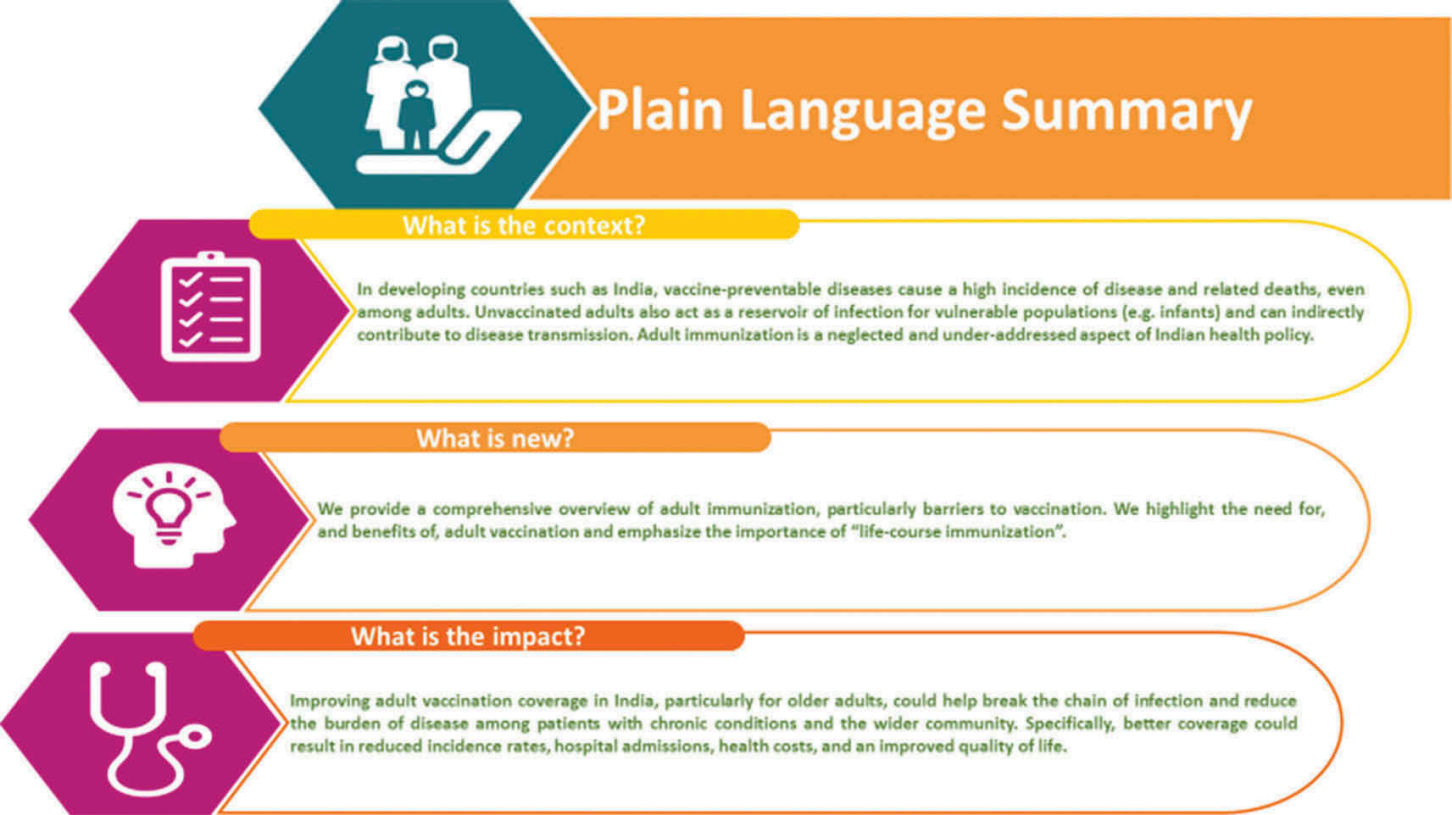
Plain language summary.

Various commentaries have highlighted the lack of adult immunization in India^15^ and the importance of improving coverage.^10,15,16^ In this focused narrative review, we combine and expand upon the information contained in these commentaries in order to create awareness about the importance of adult immunization as an integral component of “life-course immunization”. To achieve this, we sought to: (1) highlight the burden of VPDs – overall and among adults – in India; (2) summarize the various Indian adult vaccination recommendations; (3) discuss the reasons for adult VPDs (e.g. lack of adult immunization, waning immunity, immunosenescence, and epidemiological shift); and (4) propose solutions or steps to be taken for the implementation of adult immunization in view of various barriers and challenges (e.g. lack of a coordinated immunization program, lack of knowledge, and vaccine hesitancy). Figure 1 elaborates on the clinical relevance of our findings in a form that could be shared with individuals by healthcare professionals.

## Overall burden of VPDs in India

The World Health Organization (WHO) has published the number of reported cases of selected VPDs in India and other countries.^17^ While these are acknowledged to “represent only a fraction of cases”, they show some interesting trends, both over time and compared to other countries. As shown in Table 1,^1,17,18^ the numbers of reported cases of most – though not all – VPDs fell dramatically over time in India,^17^ likely due to the successful introduction of childhood immunization. However, India had a disproportionately high share of the globally reported cases of diphtheria (60%), Japanese encephalitis (44%), and tetanus (40%) in 2017, given that it is home to around 18% of the world’s population (Table 1).^1,17^ Per million population, the prevalences of diphtheria, Japanese encephalitis, and tetanus in India were 2.2–3.4-fold that of the world as a whole; while prevalences of the other VPDs were similar or less (Table 1).^1,17^ Unfortunately, the WHO VPD data^17^ are not broken down by age group, so it is unclear what proportions of VPD cases are in children and adults.

**Table 1.** Reported cases of selected VPDs.^17^

	India			Cases per million population^b^ (2017)			Vaccine coverage by dose in children (India)	
VPD	Earliest (year)	2017 (% of world cases)^a^	Highest among other countries (2017)	India	Highest among other countries	World^c^	NFHS (2015–2016)^18^	WHO (2017)^17^
Polio	18,975 (1980)	0 (0%)	74 (Syrian Arab Republic)	0	4.05 (Syrian Arab Republic)	0.01	Birth dose: 79%; doses 1–3: 91%, 86%, 73%	Dose 3: 89%
Diphtheria	39,231 (1980)	5,293 (60%)	954 (Indonesia)	3.95	24.8 (Nepal)	1.17	Doses 1–3: 90%, 86%, 78%	Pregnancy TT: 82%; doses 1, 3, 4: 92%, 89%, 81%
Neonatal tetanus	11,849 (1988)	295 (13%)	478 (Pakistan)	0.22	22.5 (Central African Republic)	0.30		
Total tetanus	45,948 (1980)	4,946 (40%)	1,057 (Philippines)	3.69	30.0 (Nepal)	1.65		
Pertussis	320,109 (1980)	23,766 (17%)	16,183 (Germany)	17.7	1,031 (Switzerland)	19.1		
Measles	114,036 (1980)	12,032 (7%)	45,107 (Democratic Republic of the Congo)	8.98	1,563 (Somalia)	23.0	Dose 1: 81%	Doses 1, 2: 90%, 80%
Rubella	1,232 (2012)	2,748 (17%)	2,111 (Philippines)	2.05	81.5 (Eritrea)	2.17	NR	Dose 1: 65%
CRS	25 (2016)	76 (9%)	532 (Indonesia)	0.06	2.02 (Indonesia)	0.11		
JE	4,017 (2007)	2,043 (44%)	1,147 (China)	1.53	8.28 (Myanmar)	0.62	NR	Dose 1: 88%

CRS, congenital rubella syndrome; JE, Japanese encephalitis; NFHS, National Family Health Survey; NR, not reported; TT, tetanus toxoid; US, United States; VPD, vaccine-preventable disease; WHO, World Health Organization.

^a^India is home to 18% of the world’s population.^1^

^b^Calculated based on reported cases^17^ and reported populations.^1^

^c^VPD cases data were not available for some countries, but the whole world population was used, so the world values are likely underestimated relative to the single country values.

According to India’s National Centre for Disease Control (NCDC), there were nearly 39,000 cases of H1N1 seasonal influenza in India in 2017, resulting in 2,270 deaths (ages not specified).^19^ Further, based on the NCDC’s Integrated Disease Surveillance Programme for epidemic-prone diseases, 990–2,679 outbreaks (ages not specified) were reported each year during 2010 to 2017.^20^ During 1st January to 4th February 2018, 133 outbreaks had already been reported, most commonly measles (28%), varicella (23%), food poisoning (14%), and acute diarrheal disease (12%).^20^

## Burden of VPDs among adults in India

There is a paucity of data on the incidence of VPDs among adults in India. However, some data on human papillomavirus (HPV) infection and cervical cancer are available. For example, random HPV testing of 890 women from India in 2009–2012 found that 12% were HPV positive and 4% were borderline positive.^21^ It has been estimated that nearly 97,000 women in India are diagnosed with cervical cancer and over 60,000 die from this disease each year, making it the second most common cancer and the second leading cause of cancer death in India.^22^

Further, data from the US Centers for Disease Control and Prevention (CDC) have indicated that, in countries with a last diphtheria booster before age 6 years, approximately half of all diphtheria cases are among those aged ≥15 years, and this was dominated by cases from India.^23^ There are also numerous reports of VPD outbreaks in India that have affected predominantly adults, some examples of which are listed in Table 2.^24-35^

**Table 2.** A selection of studies reporting outbreaks that predominantly affected adults in India.

Author	Period	VPD	Setting	State/Union territory	Adults/total
Nair et al.^24^	Apr 2005 to Mar 2006	Meningococcal disease	Tertiary care hospital	Delhi	34^a^/55
Patel et al.^25^	Feb to Aug 2009	Hepatitis B	Town	Gujarat	550^b^/664
Biswas et al.^26^	Jul to Aug 2010	Influenza A(H1N1)	City	West Bengal	104^a^/129
Peter et al.^27^	Jun to Jul 2011	Influenza A(H3N2)	District	Kerala	54^a^/96
Gurav et al.^28^	Feb to Apr 2012	Influenza A(H1N1)pdm09	Community	Maharashtra	30^c^/59
Rathi et al.^29^	Nov 2013 to Jan 2014	Measles	University	Karnataka	20^d^/20
Gurav et al.^30^	July 2014	JE	Region	West Bengal	106^c^/134
Agrawal et al.^31^	Jan to Mar 2015	Influenza A(H1N1)pdm09	Tertiary care center	Rajasthan	44 pregnant or puerperal women
Das et al.^33^	Sep 2015 to Jan 2016	Diphtheria	District	Assam	5^e^ or 7^a^/10
Meyers et al.^34^	Feb 2016 to Jan 2017	Varicella	University	Tamil Nadu	77 students (≥17 years)
Rakesh et al.^32^	Nov to Dec 2016	Hepatitis A	District	Kerala	201^d^/223
Vaidya et al.^35^	Dec 2016 to Feb 2017	Varicella	Villages	Dadra and Nagar Haveli	91^a^/149 suspected; 19^a^/31 confirmed^f^

^a^Adults defined as ≥15 years old.

^b^Adults defined as ≥21 years old.

^c^Adults defined as ≥20 years old.

^d^Adults defined as ≥16 years old.

^e^Adults defined as ≥18 years old.

^f^Only 37/149 suspected cases were tested, of which 31 (84%) were confirmed varicella.

JE, Japanese encephalitis; VPD, vaccine-preventable disease.

## Adult vaccination recommendations in India

The only nationally recommended vaccine for adults is tetanus toxoid (TT) during pregnancy – for the protection of newborns against tetanus; TT is also recommended at age 16 years.^36^ However, it should be noted that this is in the process of being replaced by tetanus and diphtheria toxoid (Td).^37,38,39^ Various Indian societies and associations have published vaccination guidelines for all adults,^40-42^ and for women^43,44^ specifically (Table 3). Although these guidelines have some similarities, there are also differences between them. Vaccination advice also depends on various, complex risk factors (e.g. chronic conditions, age, occupations, lifestyle) and vaccination/disease history. Given these discrepancies between the guidelines, it is not clear which vaccines healthy and at-risk adults should receive.

**Table 3.** Summary of adult vaccination recommendations in India.

Guidelines	Based on API^42^ and CDC^45^	ISN^40^	IMA^41^	FOGSI^43,44^
Diphtheria/tetanus/pertussis	Tdap once then Td 10-yearly; Tdap during each pregnancy	Td 10-yearly to 65 years; 3 doses if not immunized	Tdap 10–18 years (1 dose); Td 10-yearly; TT/Td early during pregnancy (2 doses); Tdap during third trimester of pregnancy (1 dose)	TT/Td 10-yearly; TT/Td or Tdap during pregnancy; Tdap if age ≥65 years, unvaccinated, and close contact with infant
MMR	19–59 years (1 or 2 doses)^a^	Unimmunized (1 dose)	Adults (1 or 2 doses)^a^	MMR for routine preconceptional/postnatal (pregnancy should be deferred for 3 months)
Influenza	Yearly (1 dose); during pregnancy (1 dose)	At risk, including during pregnancy (1 dose)^b^	Yearly (1 dose); including pregnancy	Yearly; also during pregnancy
Pneumococcal	≥65 years (1 dose); at risk^b^ (1 or 2 doses)	≥65 years (1 dose); at risk^b^	At risk^b^ (2 doses)	–
HPV	Women ≤26 years (3 doses); men ≤21 years^c^ (3 doses)	9–26 years (2 or 3 doses)	Females 9–14 years: 2 doses; 15–45 years: 3 doses	9–26 years; next dose(s) should be deferred in case of pregnancy
Varicella	2 doses if no evidence of immunity (contraindicated during pregnancy)	Nonimmune (2 doses)	Adults (2 doses)^a^	Preconception/postnatal
Herpes zoster	≥50 years (1 or 2 dose based on type of vaccine)^d^	>60 years (1 dose)	–	–
Hepatitis A	At risk^b^ (2 or 3 doses)	At risk^b^ (2 doses)	Adults (1 dose of live or 2 doses of inactivated)	–
Hepatitis B	At risk^b^ (3 doses)	At risk^b^ (3 doses)	Adults (3 doses)	Preconception^e^ or at high risk during pregnancy
Meningococcal	At risk^b^ (1 to 3 doses)	At risk^b^ (1 or 2 doses)	Adults (1 dose); At risk^b^ (2 doses)	–
Hib	At risk^b^ (1 or 3 doses)	At risk^b^ (1 dose)	At risk^b^ (1 or 3 dose)	–
Typhoid	1 dose PSV repeat 2 yearly	Outbreak or high-risk travelers (3 doses, repeat 3-yearly)	≤18 years: TCV 1 dose; >18 years: PSV 3 yearly	–
Rabies	At risk^b^ (3 or 4 doses)^b^	At risk^b^ (3 doses)	At risk^b^ (3 or 4 doses)	–
Cholera	18–65 years (1 dose if traveling to endemic region)	At risk^b^ (2 doses)	At risk^b^ (2 doses)	–
JE	2 doses; booster (in reexposure) if primary given >1 year ago	No	≤18 years (1 dose)	–
Yellow fever	1 or 2 doses based on immune status	–	At risk^b^	–
Polio	1 to 3 doses^a^	Travel to polio endemic countries (1 or 3 doses)	2 doses of IPV^a^	–
Rotavirus	–	No	–	–

^a^Based on vaccination history.

^b^The definitions of “at risk” vary by guideline and by vaccine, but include factors such as chronic health conditions, age, occupations (e.g. healthcare workers, veterinarians), lifestyles (e.g. illicit drug users, men who have sex with men), after exposure, times of epidemic, and lack of prior vaccination.

^c^ ≤ 26 years if they have sex with men.

^d^CDC does not recommend the use of Zostavax among those aged 50–59 years.

^e^Schedule completed before conception, usually 3 months prior to conception.

API, Association of Physicians of India; CDC, Centers for Disease Control and Prevention; FOGSI, Federation of Obstetric & Gynecological Societies of India; Hib, *Haemophilus influenzae* b; HPV, human papillomavirus; IMA, Indian Medical Association; ISN, Indian Society of Nephrology; JE, Japanese encephalitis; MMR, measles, mumps, rubella; PSV, Polysaccharride vaccine; TCV, Typhoid conjugate vaccine; Td, tetanus, diphtheria; Tdap, tetanus, diphtheria, acellular pertussis; TT, tetanus toxoid; US, United States.

## Reasons for VPDs in adults

There are four main reasons why adults might be susceptible to outbreaks of VPDs (Table 2): (1) lack of adult immunization; (2) waning immunity; (3) age-related factors (including immunosenescence); and (4) epidemiological shift.

### Lack of adult vaccination

#### Coverage

Adult vaccination coverage in India is generally believed to be poor,^15^ but we were unable to find reliable estimates of adult immunization coverage in India in the literature. However, some data on HPV vaccination are available. For example, in a 2014 study of 155 female healthcare professionals in Bangalore, the majority of whom had good knowledge of – and favorable attitudes toward – vaccination and screening, none had received HPV vaccination.^46^

#### Barriers to adult vaccination

**Figure 2.**
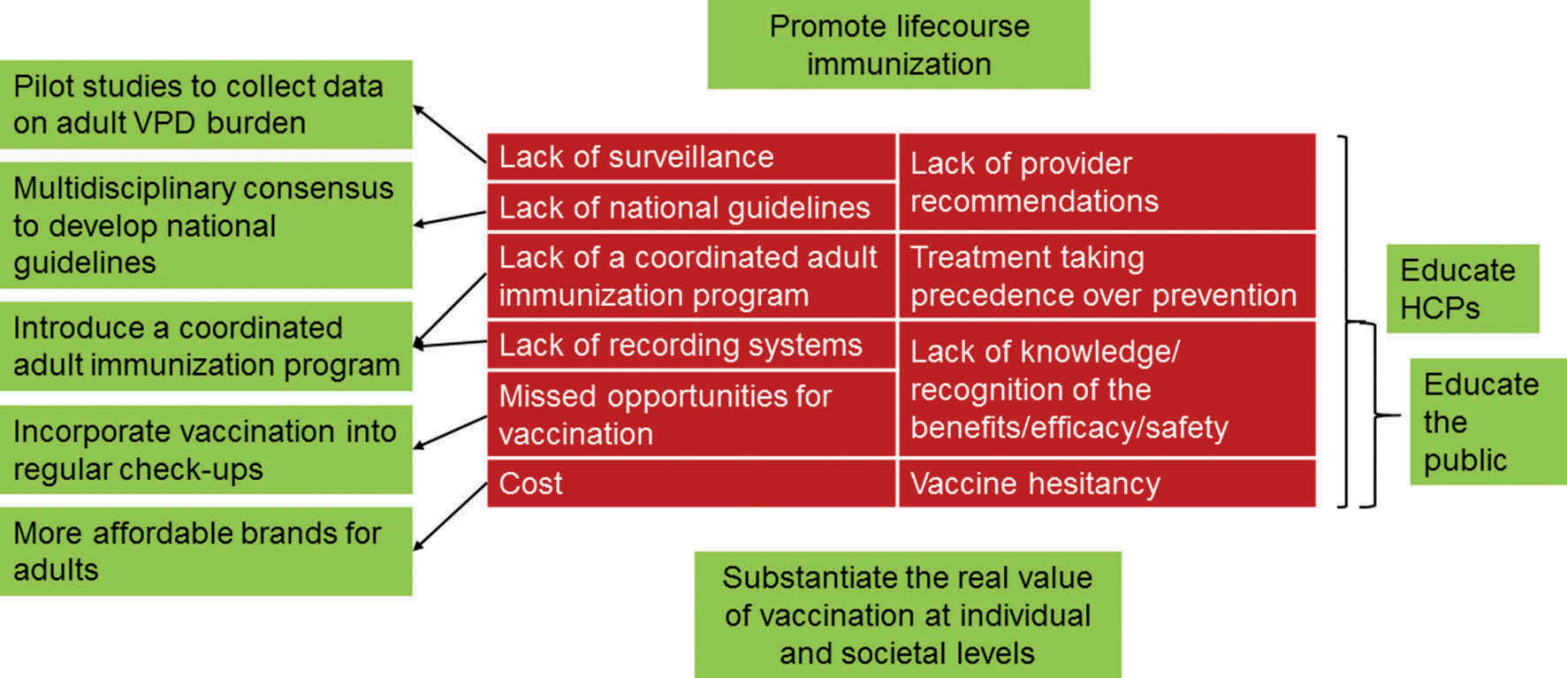
Barriers to adult vaccination (red) and next steps (green). HCP, healthcare professional; VPD, vaccine preventable disease.

Various reported barriers to adult vaccination – mainly from the US – are shown in the red sections of Figure 2.^12,47-50^ While these barriers are likely also applicable to India, there are probably a number of additional barriers relating to socio-economic factors and religious/cultural beliefs, particularly in rural India. Disease surveillance amongst Indian adults is poor, resulting in under-recognition of outbreaks and a paucity of data on the real burden of VPDs in India.^51,52^

In India, there are various adult vaccination recommendations (Table 3), but none has been nationally adopted and no systematic programs are in place to recommend, promote, or fund any of these schedules. The lack of a national commitment to improve adult vaccination has resulted in adult immunization being neglected and underpublicized. Further, over 2/3 of Indian adults are not aware of adult vaccination, many thinking that vaccines are only for children.^53^ There is also a lack of infrastructure for adult immunization in India.

Even in countries where adult vaccines are nationally recommended, uptake remains suboptimal.^54^ In the US, various age-specific vaccinations are recommended for adults.^55^ However, survey data from 2015 indicate that uptake in the US only ranged from 23% (for tetanus+pertussis; previous 10 years) to 64% (for pneumococcal disease; ever; among those aged ≥65 years).^56^ This is likely due to several factors, including “vaccine hesitancy”, which can be caused by complacency (lack of perceived need), convenience (lack of accessibility or affordability), and confidence (lack of trust) (the “3Cs” model).^57^ Recently, an additional “C” – cultural acceptance – has resulted in the “4Cs” model, because sociocultural, religious, psychological, and political factors can also affect vaccine uptake.^47,58,59^

Yearly seasonal influenza vaccination is recommended in India in various guidelines.^41-43^ Guidelines also recommend influenza vaccination during pregnancy; and it is recommended by the Indian Society of Nephrology^40^ for high-risk people, including healthcare workers (Table 3). However, in a 2012–2013 study in Srinagar, 0/1,000 pregnant women and 9/90 obstetricians had received influenza vaccination, mainly due to poor knowledge about their availability and concerns about efficacy.^60^

In an Indian interview study,^61^ 95% of respondents thought that an H1N1 influenza vaccine would be effective, but only 25% were previously aware of pandemic influenza vaccines and only 8% received one during the 2009–2010 pandemic. Barriers to vaccination included a low perceived risk, access and cost issues, insufficient information, and the perceived lack of a government mandate.^61^ Similarly, only 13% of 802 students reported receiving influenza H1N1 vaccination during the post-pandemic phase.^62^ Most (74%) said that they would definitely/probably not get vaccinated in the future, because of low perceived risk (42%) and a lack of trust in vaccine efficacy (24%) and safety (21%).

Although HPV vaccination is recommended for girls aged 9–18 years^63^ and for adults up to various ages (Table 3),^40-44^ in a survey of 210 Indian physicians, only 47% knew that HPV vaccines were approved for use in India and only 30% said they would recommend HPV vaccination.^64^ In a 2014 study of medical/paramedical students in a teaching hospital in India, only 7% had received HPV vaccination, and 49% of the unvaccinated students said that they would be unwilling to be vaccinated due to concerns about efficacy (30%), safety (26%), cost (22%), and low perceived risk (15%).^65^

The cost of vaccination – including purchase, distribution, storage, and administration costs – can affect uptake. While childhood vaccinations included in India’s Univeral Immunization Program are free of cost,^36^ the cost of adult vaccinations (apart from TT/Td during pregnancy) is currently borne by the individual.

### Waning immunity

It is well recognized that the immunity elicited by vaccines can wane over time, and that this effect is much more pronounced with vaccines against diseases caused by pathogens that have complex life cycles or antigenic variation (e.g. influenza, pertussis).^66^ This decrease in immunity years after receipt of childhood vaccines can result in outbreaks or epidemics^66^ (Table 2).

### Age-related factors

**Figure 3.**
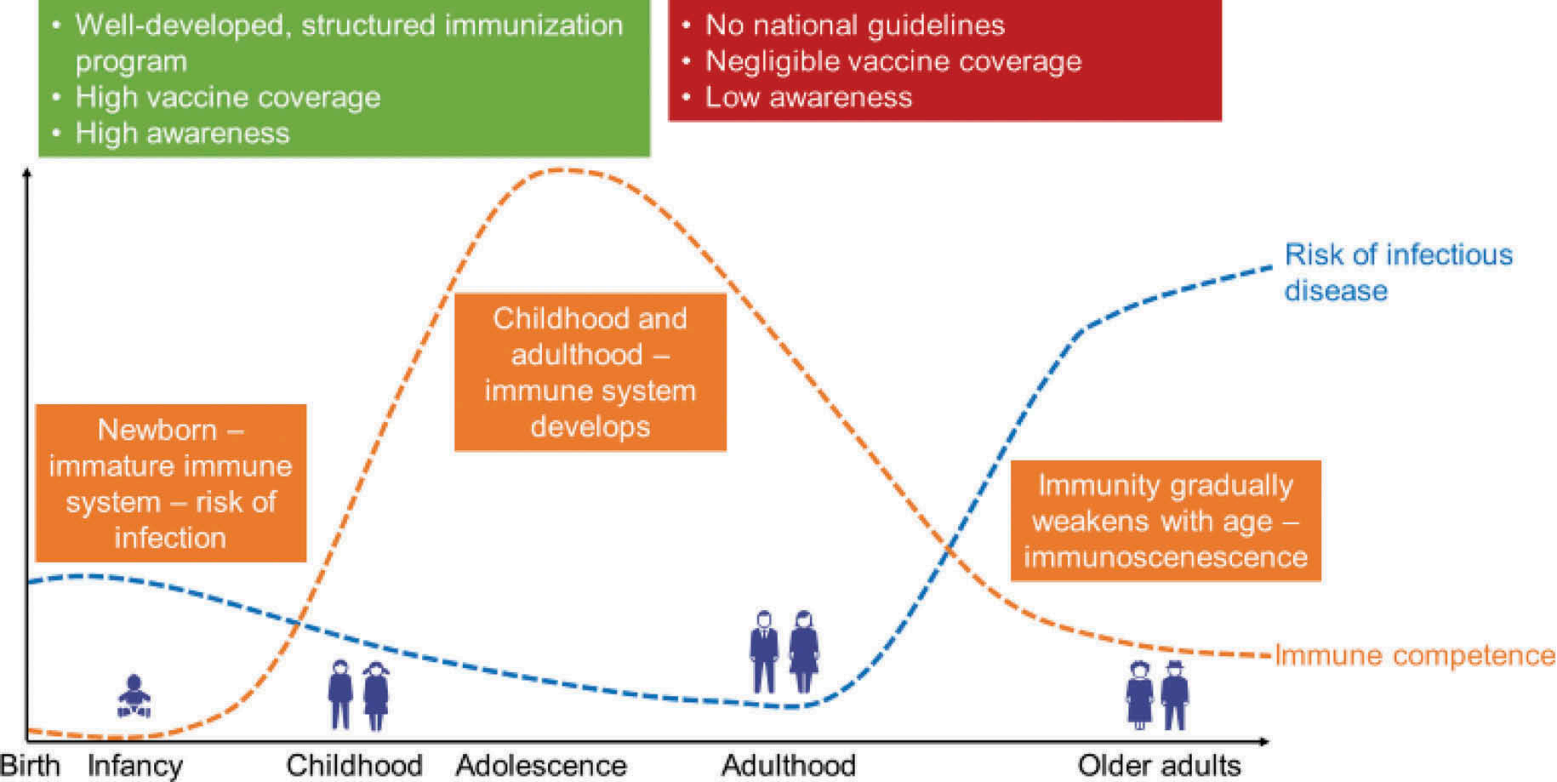
How immune competence, risk of infectious disease, immunization programs, vaccine awareness, and vaccine coverage vary with age.

Immunosenescence is a natural, progressive dysregulation and dysfunction of the innate and adaptive immune responses^47,67^ (see orange line in Figure 3). This is particularly relevant for older adults, and highlights the importance of vaccination in this age group.^47^ However, immunosenescence can alter the efficacy of some vaccines,^67^ so older adults may need different vaccines to younger adults.^47^

Various age-related conditions (e.g. obesity, chronic heart disease, stroke, diabetes, and chronic obstructive pulmonary disease) can increase the risk of VPDs and/or their complications (e.g. influenza, pneumococcal disease, and hepatitis B).^55,68,69^ Worldwide, there is an aging population, with various associated healthcare needs and challenges.^70-72^ The population in India is also aging, with the proportion of people aged ≥15 years increasing from 68% in 2007 to 72% in 2017; and the proportion of adults aged ≥65 years increasing from 5% to 6%.^6^ As the overall population ages, the burden of VPDs will likely increase.

It should also be noted that childhood immunization was only introduced in India in 1978,^73^ so anyone aged >41 years in 2019 would likely not have received any childhood vaccination. Further, vaccine coverage remained below 65% until 2014,^74^ so many Indian adults aged <41 years would also likely not have received childhood vaccinations. Adult vaccinations are even more important among these unimmunized adults.

### Epidemiological shift

Various VPDs that are normally contracted during infancy or childhood are now affecting older children and adults. This epidemiological shift could be due to increasing vaccine coverage among children. For example, in 2013–2014, there was a measles outbreak that affected 20 university students in Karnataka.^29^ Similarly, an epidemiological shift in age at diagnosis has also been reported for mumps and rubella to adolescents and adults.^75^ This is particularly important during pregnancy, as rubella infection at this time can result in congenital rubella syndrome, which is characterized by congenital organ malformations.^76^ Another example is a shift in the age of acquisition of hepatitis A virus from childhood to adolescence and early adulthood, leading to increased symptomatology and risk of liver failure.^77^ Widespread use of diphtheria-pertussis-tetanus vaccine in childhood has also been blamed for a rise in adult diphtheria and tetanus due to reduced natural boosting of immunity.^78^

## Steps to improve adult vaccination and overcome the barriers

A 2012 report for the Organisation of Pharmaceutical Producers of India reported that stakeholders should “play a significant role in protecting the lives of children, adolescents and adults, through vaccination”, but that the concept of adult vaccination in India is not established, with antibiotics or other treatments being used in adults in preference to vaccines.^79^ This report had several recommendations that could help to improve adult vaccination, including improving awareness, incorporating adult vaccinations into regular checkups, and creating more affordable vaccine brands for adults (see green sections in Figure 2).

A study from seven European countries reported that adult vaccination (as per local guidelines) was less costly than infant/childhood/adolescent vaccines.^80^ Also, that adult vaccination costs would likely be lower (on a per-person lifetime basis) than other commonly used secondary preventive measures (e.g. lipid-lowering drugs, bisphosphonates, antihypertensives, antihyperglycemics, and antithrombotics).^80^

Various studies support the cost-effectiveness adult vaccinations (with or without age or risk restrictions), but data are mainly from high-income countries.^81-84^ However, a recent study has estimated that introducing HPV vaccination among 10-year-old girls in India would cost US$15 per woman reached, $886 per cervical cancer case averted, and US$1,239 per death averted (undiscounted costs).^85^ Further investigation into the cost-effectiveness of adult vaccines in lower-middle income countries such as India would be beneficial.

Ways to improve adult vaccination coverage in India are shown in the green sections in Figure 2. Unfortunately, this will take time. Even if national guidelines for adult immunizations are adopted, promoting these guidelines and the importance of adult vaccination would require considerable effort. The inclusion of free adult vaccinations in the Universal Immunization Program would be the ultimate goal, but policymakers would have to ascertain which VPDs and which risk groups (e.g. older adults, pregnant women, healthcare professionals, patients with underlying diseases) to target initially.

### Surveillance of VPDs

Information on the adult burden of VPDs in India is lacking. Such knowledge of the Indian epidemiological profile would help in the assessment of public health needs and the impact of implementation of strategies.^86^ Pilot initiatives to collect evidence on the VPD burden in adults could be a first step to providing useful insights into the current issues and possible solutions.

### National guidelines

National guidelines would likely be based on the recommendations in Table 3. They would have to take factors such as health status, age, risk factors, and vaccination history into account. Table 4 shows which vaccines are recommended for various groups of adults in the US;^87^ a similar table could be compiled for India. National multidisciplinary coordination and leadership of adult and life-course immunization will be vital, particularly as adults tend to visit a variety of healthcare professionals.^49^

**Table 4.** Summary of adult vaccination recommendations in the United States (US) by indication. Adapted from the Advisory Committee on Immunization Practices, with permission.^87^

^a^Precaution for LAIV does not apply to alcoholism.

^b^See notes on Centers for Disease Control and Prevention website for influenza, hepatitis B, MMR, and varicella.^87^

CD4, cluster of differentiation 4-presenting immune cells; Hib, *Haemophilus influenzae* b; HIV, human immunodeficiency virus; HPV, human papillomavirus; HSCT, hematopoietic stem cell transplantation; IIV, inactivated influenza vaccine; LAIV, live attenuated influenza vaccine; MenACWY, meningococcal A, C, W, Y; Men B, meningococcal B; MMR, measles, mumps, rubella; PCV13, 13-valent pneumococcal conjugate vaccine; PPSV23, 23-valent pneumococcal polysaccharide vaccine; RIV, recombinant influenza vaccine; RZV, recombinant zoster vaccine; Td, tetanus, diphtheria; Tdap, tetanus, diphtheria, acellular pertussis; US, United States; y, years; ZVL, zoster vaccine live. ^c^Immunocompromized.

### Education

Awareness of the benefits of adult vaccination should be promoted among healthcare professionals, along with distribution of new adult immunization guidelines. Campaigns to promote awareness among the public would also be required, perhaps using cartoons or comics to attract attention and curiosity, and to demystify vaccination.^88,89^ As for current advice about a healthy diet and exercise, adult vaccination should be publicized on digital channels, at school, in the workplace, and in leisure facilities to help make them part of the social norm for a healthy lifestyle.

Pandemic influenza vaccination uptake in India has been reported to be higher among those who had seen information in the media.^61^ Door-to-door delivery of pamphlets was also useful for people to know where to go to receive the vaccine.^61^ In a study of Indian students, information about H1N1 influenza was more likely to have been received from the media (56%) or internet (41%) than from healthcare personnel (3%).^62^ In a survey of parents in Mysore, willingness to accept HPV vaccination for their daughters was improved among those who knew that cervical cancer was serious and that HPV vaccination provided protection against cervical cancer, and those who perceived HPV vaccination to be safe.^90^ These studies highlight the potential impact of education on vaccine uptake.

Healthcare workers can also contribute to increasing vaccination uptake by improving convenience, enhancing confidence, removing complacency, and being mindful of cultural acceptance (the “4Cs” model).^47^ Provider recommendation and vaccination offer are key predictors of adult vaccination, but such recommendations are often not given,^48,91^ hence providers should be encouraged to routinely assess their patient’s vaccine needs.^48^ This could also be improved by setting up provider reminders and vaccination standing orders; and reminders could also be provided to patients.^48^

### Life-course immunization

According to the WHO, a “life-course approach to health encompasses strategies across individuals’ lives that optimize their functional ability, thereby enabling well-being and the realization of rights”.^92^ This involves looking at healthcare as a continuum through life – a dynamic and interconnected process – as opposed to rigid life stages.^93^ “Life-course immunization” focusses on the extension of vaccination from birth through childhood, adolescence, adulthood, and into older age.^47,94^ This can involve catch-up vaccinations for those not immunized at the recommended ages and/or booster doses to address waning immunity.^47,94^ Figure 3 shows how the immune system improves from birth to adulthood and then declines with older age (orange line), with a concomitant decrease and then rise in the risk of infectious disease (blue line). This highlights the importance of vaccination for older adults, who become increasingly likely to have immunosenescence.^47^

## Conclusions

We fear that, if not addressed, the burden of infectious disease is likely to have far-reaching devastating effects on humanity, especially with the increase in antibiotic resistance. The creation of awareness and public education are not sufficient to bring infectious diseases under control unless they are supported by clear recommendations. We believe that efforts should focus on VPDs, as there is already an available solution to this problem. All stakeholders – including experts (researchers) and policy makers (politicians and health professionals) – should come together and ensure that the necessary steps are undertaken.

“Life-course immunization” has been adopted in many countries, as adult vaccination is an important contributor to healthy living. However, adult vaccination coverage in India is currently negligible,^15^ due to a lack of national guidelines and perceived need. This failure to immunize adults – particularly those with chronic conditions – can leave them vulnerable to infection and at increased risk of complications. Improving adult vaccination coverage in India, particularly among older adults, could help to reduce the burden of disease among those with chronic conditions by reducing hospital admissions, health costs, and mortality rates, and improving quality of life.

## References

[1] Worldometers. Countries in the world by population. [accessed 2018 18]. http://www.worldometers.info/world-population/.

[2] Berman P, Ahuja R, Bhandari L. The impoverishing effect of healthcare payments in India: new methodology and findings. Econ Polit Wkly. 2010;45:65–71.

[3] The Center For Disease Dynamics EPC. 63 million Indians are pushed into poverty by health expenses each year—and drugs are the chief cause. [accessed 2018 124]. https://cddep.org/blog/posts/63_million_indians_are_pushed_poverty_health_expenses_each_year-and_drugs_are_chief_cause/.

[4] Ministry of Health & Family Welfare. National health policy 2015 draft. [accessed 2019 327]. https://www.nhp.gov.in/sites/default/files/pdf/draft_national_health_policy_2015.pdf.

[5] Dandona L, Dandona R, Kumar GA, Shukla DK, Paul VK, Balakrishnan K, Prabhakaran D, Tandon N, Salvi S, Dash AP. India State-Level Disease Burden Initiative Collaborators. Nations within a nation: variations in epidemiological transition across the states of India, 1990-2016 in the Global Burden of Disease Study. Lancet. 2017;390:2437–60. doi:10.1016/S0140-6736(17)32804-0. 29150201 PMC5720596

[6] statista. India: age distribution from 2007 to 2017. [accessed 2018 124]. https://www.statista.com/statistics/271315/age-distribution-in-india/.

[7] Indian council of medical research, public health foundation of India, Institute for health metrics and evaluation. India: health of the nation’s states. The india state-level disease burden initiative. [accessed 2018 124]. https://www.healthdata.org/sites/default/files/files/policy_report/2017/India_Health_of_the_Nation%27s_States_Report_2017.pdf.

[8] Fonkwo PN. Pricing infectious disease. The economic and health implications of infectious diseases. EMBO Rep. 2008;9(Suppl 1):S13–7. doi:10.1038/embor.2008.110. 18578017 PMC3327542

[9] Lobanovska M, Pilla G. Penicillin’s discovery and antibiotic resistance: lessons for the Future? Yale J Biol Med. 2017;90:135–45. 28356901 PMC5369031

[10] Mehta B, Chawla S, Kumar V, Jindal H, Bhatt B. Adult immunization: the need to address. Hum Vaccin Immunother. 2014;10:306–09. doi:10.4161/hv.26797. 24128707 PMC4185890

[11] Khabbaz RF, Moseley RR, Steiner RJ, Levitt AM, Bell BP. Challenges of infectious diseases in the USA. Lancet. 2014;384:53–63. doi:10.1016/S0140-6736(14)60890-4. 24996590 PMC7137922

[12] Tan L. Adult vaccination: now is the time to realize an unfulfilled potential. Hum Vaccin Immunother. 2015;11:2158–66. doi:10.4161/21645515.2014.982998. 26091249 PMC4635860

[13] Dardis MR, Koharchik LS, Dukes S. Using the health belief model to develop educational strategies to improve pertussis vaccination rates among preschool staff. NASN Sch Nurse. 2015;30:20–25. doi:10.1177/1942602X14549256. 25626237

[14] Nieves DJ, Heininger U. Bordetella pertussis. Microbiol Spectr. 2016;4. doi:10.1128/microbiolspec.EI10-0008-2015 27337481

[15] Verma R, Khanna P, Chawla S. Adult immunization in India: importance and recommendations. Hum Vaccin Immunother. 2015;11:2180–82. doi:10.4161/hv.29342. 25483654 PMC4635930

[16] Rathi A, Sharma S. Vaccine preventable diseases in Indian adults - burden & prevention. Infect Dis Diag Treat. 2017;J102.

[17] World Health Organization (WHO). Data, statistics and graphics. [accessed 2018 12. https://www.who.int/immunization/monitoring_surveillance/data/en/.

[18] Ministry of health and family welfare. India. National Family Health Survey (NFHS-4). 2015-16. [accessed 2019 114]. http://rchiips.org/nfhs/NFHS-4Reports/India.pdf.

[19] National Centre for Disease Control. Seasonal Influenza (H1N1) – state/UT-wise, Year-wise number of cases and death from 2011 to 2018 (till 09th December, 2018). [accessed 2019 13]. https://ncdc.gov.in/showfile.php?lid=280.

[20] National Centre for Disease Control. Integrated Disease Surveillance Programme (IDSP). [accessed 2019 13]. https://ncdc.gov.in/index1.php?lang=1&level=1&sublinkid=143&lid=54.

[21] Pandey S, Mishra M, Chandrawati C. Human papillomavirus screening in North Indian women. Asian Pac J Cancer Prev. 2012;13:2643–46. doi:10.7314/APJCP.2012.13.6.2643. 22938435

[22] HPV Information Centre. India. Human papillomavirus and related cancers, fact sheet 2018. [accessed 2019 13]. http://hpvcentre.net/statistics/reports/IND_FS.pdf?t=1546505768606.

[23] Clarke KEN. Review of the epidemiology of diphtheria – 2000-2016. [accessed 2018 128]. https://www.who.int/immunization/sage/meetings/2017/april/1_Final_report_Clarke_april3.pdf.

[24] Nair D, Dawar R, Deb M, Capoor MR, Singal S, Upadhayay DJ, Aggarwal P, Das B, Samantaray JC. Outbreak of meningococcal disease in and around New Delhi, India, 2005-2006: a report from a tertiary care hospital. Epidemiol Infect 2009; 137:570–76. doi:10.1017/S0950268808001398. 18840317

[25] Patel DA, Gupta PA, Kinariwala DM, Shah HS, Trivedi GR, Vegad MM. An investigation of an outbreak of viral hepatitis B in modasa town, gujarat, India. J Glob Infect Dis. 2012;4:55–59. doi:10.4103/0974-777X.93762. 22529628 PMC3326959

[26] Biswas DK, Kaur P, Murhekar M, Bhunia R. An outbreak of pandemic influenza A (H1N1) in Kolkata, West Bengal, India, 2010. Indian J Med Res. 2012;135:529–33. 22664502 PMC3385238

[27] Peter S, Balakrishnan A, Potdar VA, Chadha MS, Jadhav SM. An outbreak of influenza A(H3N2) in Alappuzha district, Kerala, India, in 2011. J Infect Dev Ctries. 2015;9:362–67. doi:10.3855/jidc.5723. 25881524

[28] Gurav YK, Chadha MS, Tandale BV, Potdar VA, Pawar SD, Shil P, Deoshatwar AR, Aarthy R, Bhushan A. Influenza A(H1N1)pdm09 outbreak detected in inter-seasonal months during the surveillance of influenza-like illness in Pune, India, 2012-2015. Epidemiol Infect. 2017;145:1898–909. doi:10.1017/S0950268817000553. 28367767 PMC9203343

[29] Rathi P, Narendra V, Sathiya V, Kini S, Kumar A, Sana N, Rohini KVG. Measles outbreak in the adolescent population - matter of concern? J Clin Diagn Res. 2017;11:LC20–LC3. 10.7860/JCDR/2017/28619.10488 PMC5620807 28969166

[30] Gurav YK, Bondre VP, Tandale BV, Damle RG, Mallick S, Ghosh US, Nag SS. A large outbreak of Japanese encephalitis predominantly among adults in northern region of West Bengal, India. J Med Virol. 2016;88:2004–11. doi:10.1002/jmv.v88.11. 27096294

[31] Agrawal A, Agarwal S, Kumar V, Nawal CL, Mital P, Chejara R. A study of an influenza A (H1N1)pdm09 outbreak in pregnant women in Rajasthan, India. Int J Gynaecol Obstet. 2016;132:146–50. doi:10.1016/j.ijgo.2015.07.020. 26813261

[32] Rakesh PS, Mainu T, Raj A, Babu D, Rajiv M, Mohandas KS, Das A, Balasubramanian A. Investigating a community wide outbreak of hepatitis A in Kerala, India. J Family Med Prim Care. 2018;7:1537–41. doi:10.4103/jfmpc.jfmpc_127_18. 30613555 PMC6293930

[33] Das PP, Patgiri SJ, Saikia L, Paul D. Recent outbreaks of diphtheria in Dibrugarh District, Assam, India. J Clin Diagn Res. 2016(10):DR01–3. 10.7860/JCDR/2016/20212.8144 PMC5020183 27630847

[34] Meyers J, Logaraj M, Ramraj B, Narasimhan P, MacIntyre CR. Epidemic varicella zoster virus among university students, India. Emerg Infect Dis. 2018;24:366–69. doi:10.3201/eid2402.170659. 29350152 PMC5782884

[35] Vaidya SR, Tilavat SM, Kumbhar NS, Kamble MB. Chickenpox outbreak in a tribal and industrial zone from the union territory of Dadra and Nagar Haveli, India. Epidemiol Infect. 2018;146:476–80. doi:10.1017/S0950268818000201. 29436318 PMC9134548

[36] Ministry of Health & Family Welfare. Universal Immunization Programme (UIP). [accessed 2019 57]. https://mohfw.gov.in/majorprogrammes/Non-Communicable-Diseases-Injury-%26-Trauma/universal-immunization-programme-uip.

[37] Jayachandran N. India moves to Tetanus-Diphtheria vaccine, instead of only Tetanus, on WHO guidance. [accessed 2019 215]. https://www.thenewsminute.com/article/india-moves-tetanus-diphtheria-vaccine-instead-only-tetanus-who-recommendation-86614.

[38] World Health Organization. UNICEF. Replacement of TT with Td vaccine for dual protection. [accessed 2019 2 15]. https://www.who.int/immunization/diseases/tetanus/QA_TT_to_Td_Replacement_Final_28June2018.pdf.

[39] World Health Organization. Maternal and Neonatal Tetanus Elimination (MNTE). [accessed 2019 215]. https://www.who.int/immunization/diseases/MNTE_initiative/en/index1.html.

[40] Guidelines for vaccination in normal adults in India. Indian J Nephrol. 2016;26(1):S1–S30.

[41] Indian Medical Association. Life course immunization guidebook. A Quick Reference Guide. [accessed 2019 4 5]. http://www.ima-india.org/ima/pdfdata/IMA_LifeCourse_Immunization_Guide_2018_DEC21.pdf.

[42] Ramasubramanian V. Chapter 6. Adult Immunization in India. [accessed 2019 4 5]. http://www.apiindia.org/pdf/progress_in_medicine_2017/mu_06.pdf.

[43] Wagh G, Kurian R. Vaccination in women. [accessed 2018 124]. https://www.fogsi.org/wp-content/uploads/2015/11/vaccination_women.pdf.

[44] Federation of Obstetric & Gynaecological Societies of India (FOGSI). Good clinical practice recommendations on preconception care. [accessed 2019 55]. https://www.fogsi.org/gcpr-preconception-care/.

[45] Centers for Disease Control and Prevention (CDC). Vaccines by disease. [accessed 2019 523]. https://www.cdc.gov/vaccines/vpd/vaccines-diseases.html.

[46] Swapnajaswanth M, Suman G, Suryanarayana SP, Murthy NS. Perception and practices on screening and vaccination for carcinoma cervix among female healthcare professional in tertiary care hospitals in Bangalore, India. Asian Pac J Cancer Prev. 2014;15:6095–98. doi:10.7314/APJCP.2014.15.15.6095. 25124579

[47] Philip RK, Attwell K, Breuer T, Di Pasquale A, Lopalco PL. Life-course immunization as a gateway to health. Expert Rev Vaccines. 2018;17:851–64. doi:10.1080/14760584.2018.1527690. 30350731

[48] Bridges CB, Hurley LP, Williams WW, Ramakrishnan A, Dean AK, Groom AV. Meeting the challenges of immunizing adults. Vaccine. 2015;33(Suppl 4):D114–20. doi:10.1016/j.vaccine.2015.09.054. 26615170

[49] National Vaccine Advisory Committee. A pathway to leadership for adult immunization: recommendations of the national vaccine advisory committee: approved by the national vaccine advisory committee on June 14, 2011. Public Health Rep. 2012;127(1):1–42. doi:10.1177/00333549121270S101. PMC3235599 22210957

[50] Government of Canada. Page 2: Canadian immunization guide: part 3 - vaccination of specific populations. [accessed 2019 717]. https://www.canada.ca/en/public-health/services/publications/healthy-living/canadian-immunization-guide-part-3-vaccination-specific-populations/page-2-immunization-of-adults.html.

[51] John TJ, Dandona L, Sharma VP, Kakkar M. Continuing challenge of infectious diseases in India. Lancet. 2011;377:252–69. doi:10.1016/S0140-6736(10)61265-2. 21227500

[52] Bagcchi S. India tackles vaccine preventable diseases. Lancet Infect Dis. 2015;15:637–38. doi:10.1016/S1473-3099(15)00009-2. 26008840

[53] Aggarwal KK. Majority of Indians are unaware of adult vaccinations. [accessed 2019 57]. http://blogs.kkaggarwal.com/2017/11/vaccinations.

[54] La EM, Trantham L, Kurosky SK, Odom D, Aris E, Hogea C. An analysis of factors associated with influenza, pneumoccocal, Tdap, and herpes zoster vaccine uptake in the US adult population and corresponding inter-state variability. Hum Vaccin Immunother. 2018;14:430–41. doi:10.1080/21645515.2017.1403697. 29194019 PMC5806688

[55] Centers for Disease Control and Prevention (CDC). What vaccines are recommended for you. [accessed 2018 12]. https://www.cdc.gov/vaccines/adults/rec-vac/index.html.

[56] Williams WW, Lu PJ, O’Halloran A, Kim DK, Grohskopf LA, Pilishvili T, Skoff TH, Nelson NP, Harpaz R, Markowitz LE, et al. Surveillance of vaccination coverage among adult populations - United States, 2015. MMWR Surveill Summ. 2017;66:1–28. doi:10.15585/mmwr.ss6611a1. PMC5829683 28472027

[57] MacDonald NE. The sage working group on vaccine hesitancy. Vaccine hesitancy: definition, scope and determinants. Vaccine. 2015;33:4161–64. doi:10.1016/j.vaccine.2015.04.036. 25896383

[58] Larson HJ, Cooper LZ, Eskola J, Katz SL, Ratzan S. Addressing the vaccine confidence gap. Lancet. 2011;378:526–35. doi:10.1016/S0140-6736(11)60678-8. 21664679

[59] Ozawa S, Stack ML. Public trust and vaccine acceptance - international perspectives. Hum Vaccin Immunother. 2013;9:1774–78. doi:10.4161/hv.24961. 23733039 PMC3906280

[60] Koul PA, Bali NK, Ali S, Ahmad SJ, Bhat MA, Mir H, Akram S, Khan UH. Poor uptake of influenza vaccination in pregnancy in northern India. Int J Gynaecol Obstet. 2014;127:234–37. doi:10.1016/j.ijgo.2014.05.021. 25085688

[61] Sundaram N, Purohit V, Schaetti C, Kudale A, Joseph S, Weiss MG. Community awareness, use and preference for pandemic influenza vaccines in Pune, India. Hum Vaccin Immunother. 2015;11:2376–88. doi:10.1080/21645515.2015.1062956. 26110454 PMC4635903

[62] Suresh PS, Thejaswini V, Rajan T. Factors associated with 2009 pandemic influenza A (H1N1) vaccination acceptance among university students from India during the post-pandemic phase. BMC Infect Dis. 2011;11:205. doi:10.1186/1471-2334-11-205. 21798074 PMC3161886

[63] Balasubramanian S, Shah A, Pemde HK, Chatterjee P, Shivananda S, Guduru VK, Soans S, Shastri D, Kumar R. Indian Academy of Pediatrics (IAP) Advisory Committee on Vaccines and Immunization Practices (ACVIP) Recommended Immunization Schedule (2018-19) and update on immunization for children aged 0 through 18 years. Indian Pediatr. 2018;55:1066–74. doi:10.1007/s13312-018-1444-8. 30745480

[64] Canon C, Effoe V, Shetty V, Shetty AK. Knowledge and attitudes towards Human Papillomavirus (HPV) among academic and community physicians in Mangalore, India. J Cancer Educ. 2017;32:382–91. doi:10.1007/s13187-016-0999-0. 26880357

[65] Swarnapriya K, Kavitha D, Reddy GM. Knowledge, attitude and practices regarding HPV vaccination among medical and para medical in students, India a cross sectional study. Asian Pac J Cancer Prev. 2015;16:8473–77. doi:10.7314/APJCP.2015.16.18.8473. 26745104

[66] Gu XX, Plotkin SA, Edwards KM, Sette A, Mills KHG, Levy O, Sant AJ, Mo A, Alexander W, Lu KT, et al. Waning immunity and microbial vaccines–workshop of the national institute of allergy and infectious diseases. Clin Vaccine Immunol. 2017;24:e00034-17. doi:10.1128/CVI.00034-17 PMC5498725 28490424

[67] Goronzy JJ, Weyand CM. Understanding immunosenescence to improve responses to vaccines. Nat Immunol. 2013;14:428–36. doi:10.1038/ni.2588. 23598398 PMC4183346

[68] Torres A, Blasi F, Dartois N, Akova M. Which individuals are at increased risk of pneumococcal disease and why? Impact of COPD, asthma, smoking, diabetes, and/or chronic heart disease on community-acquired pneumonia and invasive pneumococcal disease. Thorax. 2015;70:984–89. doi:10.1136/thoraxjnl-2015-206780. 26219979 PMC4602259

[69] Green WD, Beck MA. Obesity Impairs the Adaptive Immune Response to Influenza Virus. Ann Am Thorac Soc. 2017;14:S406–S9. doi:10.1513/AnnalsATS.201706-447AW. 29161078 PMC5711276

[70] Christensen K, Doblhammer G, Rau R, Vaupel JW. Ageing populations: the challenges ahead. Lancet. 2009;374:1196–208. doi:10.1016/S0140-6736(09)61460-4. 19801098 PMC2810516

[71] Doyle Y, McKee M, Rechel B, Grundy E. Meeting the challenge of population ageing. BMJ. 2009;339:b3926. doi:10.1136/bmj.b3926. 19805472

[72] Rechel B, Grundy E, Robine JM, Cylus J, Mackenbach JP, Knai C, McKee M. Ageing in the European Union. Lancet. 2013;381:1312–22. doi:10.1016/S0140-6736(12)62087-X. 23541057

[73] Vashishtha VM. National vaccine policy of India. [accessed 2018 1213]. https://www.researchgate.net/publication/275153455_National_Vaccine_Policy_of_India.

[74] Government of India. Mission Indradhanush. [accessed 2018 125]. http://ipa-world.org/society-resources/code/images/349bc28-Mission%20Indradhanush%20Concept%20Note.pdf.

[75] Banerjee A. Outbreaks of rubella indicate epidemiological shift in age. Indian Pediatr. 2015;52:169. doi:10.1007/s13312-015-0598-x. 25691203

[76] Bhatnagar N, Kaur R, Gupta M, Sharma D. Introducing combined measles, mumps and rubella vaccine in Chandigarh, India: issues and concerns. Indian Pediatr. 2014;51:441–43. doi:10.1007/s13312-014-0428-6. 24986275

[77] Mathur P, Arora NK. Epidemiological transition of hepatitis A in India: issues for vaccination in developing countries. Indian J Med Res. 2008;128:699–704. 19246792

[78] Saxena S, Jais M, Dutta R, Dutta AK. Serological immunity to diphtheria and tetanus in healthy adults in Delhi, India. Trop Doct. 2009;39:160–63. doi:10.1258/td.2008.080274. 19535754

[79] Bhadoria V, Gobinath A, Mitra P, Narayan M. Transforming India’s vaccine market. Saving lives, creating value. [accessed 2018 128]. https://www.mckinsey.com/~/media/mckinsey/featured%20insights/india/transforming%20indias%20vaccine%20market/transforming%20indias%20vaccine%20market%20saving%20lives%20creating%20valuesept2012.ashx.

[80] Ethgen O, Cornier M, Chriv E, Baron-Papillon F. The cost of vaccination throughout life: a western European overview. Hum Vaccin Immunother. 2016;12:2029–37. doi:10.1080/21645515.2016.1154649. 27050111 PMC4994732

[81] Newall AT, Scuffham PA, Kelly H, Harsley S, Macintyre CR. The cost-effectiveness of a universal influenza vaccination program for adults aged 50-64 years in Australia. Vaccine. 2008;26:2142–53. doi:10.1016/j.vaccine.2008.01.050. 18343537

[82] Lee GM, Riffelmann M. Wirsing von Konig CH. Cost-effectiveness of adult pertussis vaccination in Germany. Vaccine. 2008;26:3673–79. doi:10.1016/j.vaccine.2008.04.068. 18538901

[83] Leidner AJ, Murthy N, Chesson HW, Biggerstaff M, Stoecker C, Harris AM, Acosta A, Dooling K, Bridges CB. Cost-effectiveness of adult vaccinations: A systematic review. Vaccine. 2019;37:226–34. doi:10.1016/j.vaccine.2018.11.056. 30527660 PMC6545890

[84] Mangen MJ, Rozenbaum MH, Huijts SM, van Werkhoven CH, Postma DF, Atwood M, van Deursen AM, van der Ende A, Grobbee DE, Sanders EA, et al. Cost-effectiveness of adult pneumococcal conjugate vaccination in the Netherlands. Eur Respir J. 2015;46:1407–16. doi:10.1183/13993003.00325-2015. 26160871 PMC4750466

[85] Campos NG, Sharma M, Clark A, Lee K, Geng F, Regan C, Kim J, Resch S. The health and economic impact of scaling cervical cancer prevention in 50 low- and lower-middle-income countries. Int J Gynaecol Obstet. 2017;138(Suppl 1):47–56. doi:10.1002/ijgo.12184. 28691334

[86] Gupte MD, Ramachandran V, Mutatkar RK. Epidemiological profile of India: historical and contemporary perspectives. J Biosci. 2001;26:437–64. doi:10.1007/BF02704746. 11779959

[87] Centers for Disease Control and Prevention (CDC). Table 2. Recommended adult immunization schedule by medical condition and other indications, United States, 2019. [accessed 2019 411]. https://www.cdc.gov/vaccines/schedules/hcp/imz/adult-conditions.html#note-zoster

[88] Muzumdar JM, Pantaleo NL. Comics as a medium for providing information on adult immunizations. J Health Commun. 2017;22:783–91. doi:10.1080/10810730.2017.1355418. 28901823

[89] Diamond J, McQuillan J, Spiegel AN, Hill PW, Smith R, West J, Wood C. Viruses, Vaccines and the public. Mus Soc Issues. 2016;11:9–16. doi:10.1080/15596893.2016.1131099. 27524953 PMC4980086

[90] Madhivanan P, Li T, Srinivas V, Marlow L, Mukherjee S, Krupp K. Human papillomavirus vaccine acceptability among parents of adolescent girls: obstacles and challenges in Mysore, India. Prev Med. 2014;64:69–74. doi:10.1016/j.ypmed.2014.04.002. 24732716

[91] Lu PJ, Srivastav A, Amaya A, Dever JA, Roycroft J, Kurtz MS, O’Halloran A, Williams WW. Association of provider recommendation and offer and influenza vaccination among adults aged ≥18 years - United States. Vaccine. 2018;36:890–98. doi:10.1016/j.vaccine.2017.12.016. 29329685

[92] Kuruvilla S, Sadana R, Montesinos EV, Beard J, Vasdeki JF, Araujo de Carvalho I, Thomas RB, Drisse MB, Daelmans B, Goodman T, et al. A life-course approach to health: synergy with sustainable development goals. Bull World Health Organ. 2018;96:42–50. doi:10.2471/BLT.17.198358. 29403099 PMC5791871

[93] World Health Organization (WHO). World report on ageing and health. 2015 [accessed 2018 124]. http://www.who.int/ageing/events/world-report-2015-launch/en/.

[94] Aguado T, Goodwin J, Holt D, Larson H, Nye S, Salisbury D, Votta M, Wilkinson J, Evans A, Wait S. A life-course approach to vaccination: adapting European policies. [accessed 2018 127]. http://www.comomeningitis.org/media/131769/life_course_vacc_policy_report_interactive-1-.pdf.

